# Supplementary material for: Immune Priming Triggers Cell Wall Remodeling and Increased Resistance to Halo Blight Disease in Common Bean
Source: Plants (Basel). 2021 Jul 23;10(8):1514. doi: 10.3390/plants10081514 (PMC8401974; doi:10.3390/plants10081514)
Supplement: Supplementary file 1 [file plants-10-01514-s001.zip › plants-1300387 Supplementary .pdf]

Supplementary figure

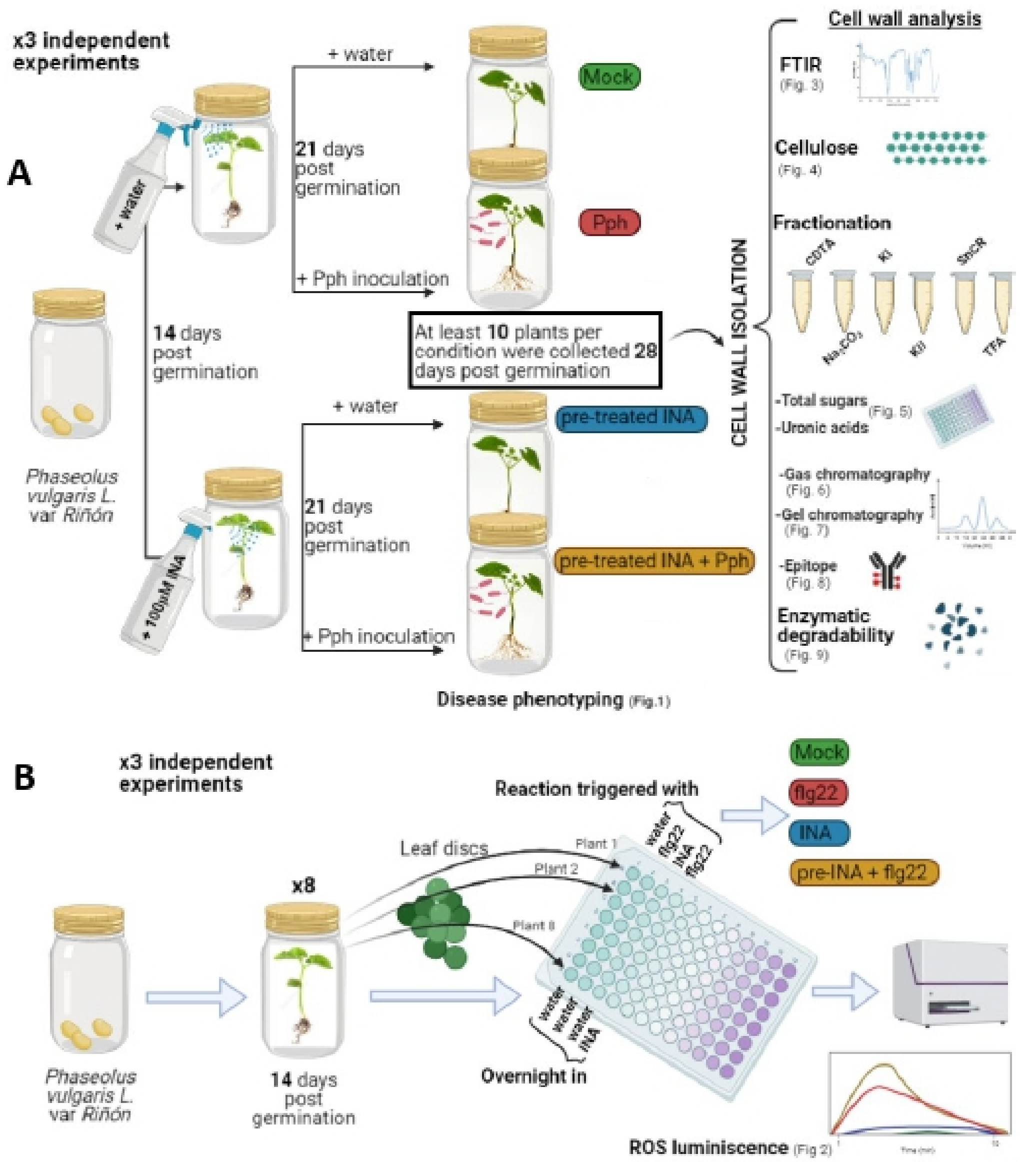

Figure S1. Experimental design. (A) Seeds of common bean *P. vulgaris* L. cultivar *Riñón* were grown *in vitro*. Leaves from common bean plants at V1 stage were sprayed with 2 mL of 100 µM INA (INA condition) per leaf or with sterilized water (Mock condition). After 7 days, some plants previously treated or not with INA were sprayed with 2 mL of the Pph solution on foliage leaves, resulting in INA + Pph and Mock + Pph treatments. All plants were grown for 7 days more and then, the foliage leaves of 10 plants per treatment were collected. The experiment was repeated three independent times (n=3). Afterwards, CWs were isolated, fractionated and their components analyzed by means of different techniques. (B) The ROS production was determined in V1 plants leaf disks. The reactions were triggered by adding 100 µL water (Mock condition) as negative control, 100 µL 200 µM INA (INA condition) as negative control for toxicity, 100 µL 2 µM flg22 (flg22 condition) as positive control, and also 100 µL 2 µM flg22 to those disks previously incubated overnight with 100 µM INA (preINA+flg22) as experimental condition. The experiment, with 8 plants assayed, was repeated three independent times (n=3).
